# Formulation Design and Optimization of Fast Dissolving Clonazepam Tablets

**DOI:** 10.4103/0250-474X.58189

**Published:** 2009

**Authors:** S. B. Shirsand, Sarasija Suresh, P. V. Swamy

**Affiliations:** Department of Pharmaceutics, H. K. E. Society's College of Pharmacy, Sedam Road, Gulbarga-585 105, India; 1 Department of Pharmaceutics, Al-Ameen College of Pharmacy, Near Lal Bagh Main Gate, Hosur Road, Bangalore-560 027, India

**Keywords:** 3^2^ full factorial design, clonazepam, crospovidone, fast dissolving tablets, microcrystalline cellulose

## Abstract

Fast dissolving tablets of clonazepam were prepared by direct compression method with a view to enhance patient compliance. A 3^2^ full factorial design was applied to investigate the combined effect of two formulation variables: amount of crospovidone and microcrystalline cellulose. Crospovidone (2-8% w/w) was used as superdisintegrant and microcrystalline cellulose (20-40% w/w) was used as diluent, along with directly compressible mannitol to enhance mouth feel. The tablets were evaluated for hardness, friability, thickness, drug content uniformity, *in vitro* dispersion time, wetting time and water absorption ratio. Based on *in vitro* dispersion time (approximately 16 s); the formulation containing 2% w/w crospovidone and 40% w/w microcrystalline cellulose was found to be promising and tested for *in vitro* drug release pattern (in pH 6.8 phosphate buffer). Short-term stability (at 40°/75% relative humidity for 3 mo) and drug-excipient interaction. Surface response plots are presented to graphically represent the effect of independent variables on the invitro dispersion time. The validity of the generated mathematical model was tested by preparing two extra-design checkpoints. The optimized tablet formulation was compared with conventional commercial tablet formulation for drug release profiles. This formulation showed nearly five-fold faster drug release (t_50%_ 3.5 min) compared to the conventional commercial tablet formulation (t_50%_ 16.4 min). Short-term stability studies on the formulation indicated that there are no significant changes in drug content and in vitro dispersion time (*P*<0.05).

Dysphagia is a common problem encountered in all age groups in concern to solid dosage forms, which results in high incidence of non-compliance and ineffective therapy[1]. Recent advances in novel drug delivery systems (NDDS) aim to enhance safety and efficacy of drug molecule by formulating a convenient dosage form for administration and to achieve better patient compliance i.e., one, which will rapidly disintegrate in the mouth without need of water (fast dissolving tablet, FDT). Advantages of this drug delivery system include administration without water, accuracy of dosage, easy portability, alternative to liquid dosage forms, ideal for pediatric and geriatric patients and rapid onset of action[2–4]. Clonazepam (CZ) is a benzodiazepine derivative with marked antiepileptic properties. It may be used in the treatment of all types of epilepsy and seizures[5]. Since epileptic patients have to strictly follow the dosage regimen for preventing sub-therapeutic concentration, FDT will avoid missing out of a dose even during travelling or other situations, where there is no access to water; offers a suitable and practical approach in serving desired objective of faster disintegration and dissolution characteristics with increased bioavailability. Aim of the present study was to develop such a NDDS for CZ by simple and cost-effective direct compression method.

CZ and crospovidone (CP) were gift samples from Torrent Pharma, Ahmedabad, India and Wockhardt Research Centre, Aurangabad, India respectively. Directly compressible mannitol (Pearlitol SD200), sodium stearyl fumarate (SSF) and microcrystalline cellulose (MCC, Avicel PH-102) were generous gifts from Strides Arco Labs, Bangalore, India, Glenmark Ltd., Nashik, India and Alkem Labs Pvt Ltd, Mumbai, India. All other chemicals were of analytical reagent grade.

After preliminary studies the formulations were designed according to the 3^2^ full factorial design, allowing a simultaneous evaluation of the two formulation variables and their interaction. The experimental design with the corresponding formulation is outlined in Table 1. The effect of the independent variables, viz., crosspovidone (X_1_) and microcrystalline cellulose (X_2_) on the dependent variable, *in vitro* dispersion time (y_1_) was evaluated. The preliminary studies indicated that the super-disintegrant CP was found to be superior compared to the other two super-disintegrants (croscarmellose sodium and sodium starch glycolate); and hence it was selected as the super-disintegrant of choice for the present investigation.

**TABLE 1:** FACTORIAL DESIGN FORMULATIONS OF CLONAZEPAM PREPARED BY DIRECT COMPRESSION METHOD

Ingredients* (mg)	Formulation Code											
	
	F1	F2	F3	F4	F5	F6	F7	F8	F9	F0	C1	C2
Clonazepam	2	2	2	2	2	2	2	2	2	2	2	2
Crospovidone	3	3	3	7.5	7.5	7.5	12	12	12	--	5.25	9.75
MCC	30	45	60	30	45	60	30	45	60	30	37.5	52.5
Aspartame	3	3	3	3	3	3	3	3	3	3	3	3.
Sodium steady fumarate	1.5	1.5	1.5	1.5	1.5	1.5	1.5	1.5	1.5	1.5	1.5	1.5
Flavor (pineapple)	1.5	1.5	1.5	1.5	1.5	1.5	1.5	1.5	1.5	1.5	1.5	1.5
Talc	3.0	3.0	3.0	3.0	3.0	3.0	3.0	3.0	3.0	3.0	3.0	3.0
Directly compressible mannitol	106	91	76	101.5	86.5	71.5	97.0	82.0	67.0	109.0	96.25	76.75

* All the quantities expressed are in mg/tablet. Formulation F3 was selected as the best and used in further studies. F0 is control formulation; C1 and C2 are extra design check-point formulations.

Fast dissolving tablets of CZ were prepared by direct compression method[6] according to the formulae given in Table 1. All the ingredients were passed through #60 mesh separately, weighed and mixed in geometrical order. Then lubricant and glidant (# 200 mesh) were added and mixed for further 5 min. The blend thus obtained was directly compressed using 7 mm flat round punches into tablets of 150 mg on a 10-station rotary tablet machine (Clit, Ahmedabad, India). A batch of 60 tablets was prepared for all the designed formulations.

For assessing weight variation twenty tablets were selected at random and assessed individually. The individual weights were compared with the average weight for determination of weight variation[7]. Hardness and friability of the tablets were determined by using Monsanto hardness tester and Roche friabilator, respectively. For content uniformity test, 10 tablets were weighed and powdered, a quantity of powder equivalent to 2 mg of CZ was extracted into methanol and liquid was filtered. The CZ content was determined by measuring the absorbance at 308 nm after appropriate dilution with methanol. The drug content was determined using the standard calibration curve. The mean percent drug content was calculated using 3 determinations[8]. For determination of *in vitro* dispersion time, 1 tablet was placed in a beaker containing 10 ml of pH 6.8 phosphate buffer at 37±0.5° and the time required for complete dispersion was determined[9]. For determination of wetting time and water absorption ratio[10], a piece of tissue paper folded twice was placed in a small 5 cm diameter Petri dish containing 6 ml of water. A tablet was placed on the paper and the time required for complete wetting was measured. The wetted tablet was then weighed. Water absorption ratio ‘R’ was determined using the equation, R=100(W_b_-W_a_)/W_a_; where W_a_ is weight of tablet before water absorption and W_b_ is weight of tablet after water absorption. The results are shown in Table 2. IR spectra of the pure drug and its formulations were obtained by potassium bromide pellet method using Perkin-Elmer FTIR series (model 1615) spectrophotometer in order to rule out drug-carrier interactions.

**TABLE 2:** EVALUATION OF FACTORIAL DESIGN FDT FORMULATIONS

Parameter	Formulation code									
	
	F0	F1	F2	F3	F4	F5	F6	F7	F8	F9
Hardness* (kg/cm_2_) ±SD	2.50±0.10	2.46±0.057	2.50±0.10	2.70±0.10	2.80±0.10	2.46± 0.057	2.42±0.072	2.96±0.10	2.83±0.152	2.70±0.10
Friability (%)	0.52	0.45	0.40	0.46	0.45	0.42	0.48	0.50	0.52	0.45
Thickness (mm)	3.60	3.55	3.57	3.52	3.15	3.40	3.60	3.20	3.50	3.40
*In vitro* dispersion time* (seconds) ±SD	86.0±1.52	53.33±1.52	28.00±1.00	16.00±2.0	24.30±1.52	20.00±1.00	11.60±1.52	16.60±0.57	9.00±1.00	7.33±0.57
Wetting time* (seconds) ±SD	90.0±1.00	56.00±2.00	30.60±2.08	17.58±1.14	26.00±1.00	22.00±1.23	13.00±1.00	17.33±0.70	10.30±0.57	8.00±1.00
Water absorption ratio* (%) ±SD	52.2±1.53	62.93±1.51	64.95±1.53	70.00±2.00	74.90±1.20	79.85±2.25	76.76±1.22	82.00±2.42	84.13±2.40	89.16±2.14
Percent drug content* ±SD	99.1±0.90	100.52±1.98	99.25±0.54	100.52±2.10	97.30±2.65	99.16±0.92	97.69±1.42	101.64±1.43	102.02±1.43	96.34±1.42

* Average of three determinations. Weight variation (147-155 mg) within the IP limits of ±7.5%

*In vitro* dissolution of the formulated fast dissolving tablets of CZ and one commercial conventional tablet was studied in USP XXIII type-2 dissolution apparatus (Electrolab, Model-TDT 06N) employing a paddle stirrer at 50 rpm using 900 ml of pH 6.8 phosphate buffer at 37±0.5° as dissolution medium[11]. One tablet was used in each test. Aliquots of dissolution medium (5 ml) were withdrawn at specific intervals of time and analyzed for drug content by measuring the absorbance at 307.5 nm. The volume withdrawn at each time interval was replaced with fresh quantity of dissolution medium. Cumulative percent CZ released was calculated and plotted against time.

Short-term stability studies on the optimized promising formulation (F_3_) were carried out by storing the tablets (in amber colored rubber stoppered vials) at 40°/ 75% RH for 3 mo period (as per ICH guidelines). At intervals of 1 mo, the tablets were visually examined for any physical changes, changes in drug content and *in vitro* dispersion time. A 3^2^ randomised full factorial design was adopted to optimize the variables. In this design 2 factors were evaluated, each at 3 levels, and experimental trials were performed at all nine possible combinations[12]. The amounts of crospovidone (X_1_), and microcrystalline cellulose (X_2_), were selected as independent variables. *In vitro* dispersion time was selected as dependent variable/response (Y_1_).

Fast dissolving tablets of CZ were prepared by direct compression method using CP as super-disintigrant and MCC as diluent along with directly compressible mannitol (Pearlitol SD 200), which was used to enhance the mouth feel. A total of nine formulations and a control formulation (F_0_, without super-disintegrant) were designed.

As the material was free flowing (angle of repose value <30° and Carr's index <15%), tablets obtained were of uniform weight (due to uniform die fill), with acceptable variation as per IP specifications (±7.5%). Drug content was found to be in the range of 96-102%, which is within acceptable limits. Hardness of the tablets was found to be 2.4 to 3.0 kg/cm^2^. Friability below 1% was an indication of good mechanical resistance of the tablets (Table 2). Formulation F_3_ was found to be promising and displayed an *in vitro* dispersion time of 16 s, which facilitates faster dispersion in the mouth.

In order to investigate the factors systematically, a factorial design was employed in the present investigation. Formulation optimization has been done by using 3^2^ full factorial design, preparing nine batches of formulations (F_1_ to F_9_). A polynomial equation was derived for *in vitro* dispersion time, by backward stepwise linear regression analysis, using PCP Disso 2000 V3 software. Formulation F_3_ containing 2% w/w CP, 40% w/w MCC was found to be promising with an *in vitro* dispersion time of 16 s against the 86 s displayed by control formulation (F_0_), which does not contain the super-disintegrant CP.

*In vitro* dissolution studies on the promising formulation (F_3_), the control (F_0_) and commercial conventional tablet formulation (CF) were carried out in pH 6.8 phosphate buffer and the various dissolution parameter values, viz., percent drug dissolved in 5 min (D_5_), 10 min (D_10_), dissolution efficiency[13] at 10 min (DE_10min_), t_50%_, t_70%_ and t_90%_ are shown in Table 3 and the dissolution profiles depicted in fig. 1. This data reveals that overall, the formulation F_3_ has shown nearly five-fold faster drug release (t_50%_ 3.5 min) when compared to CF (t_50%_ 16.4 min).

**Fig. 1:**
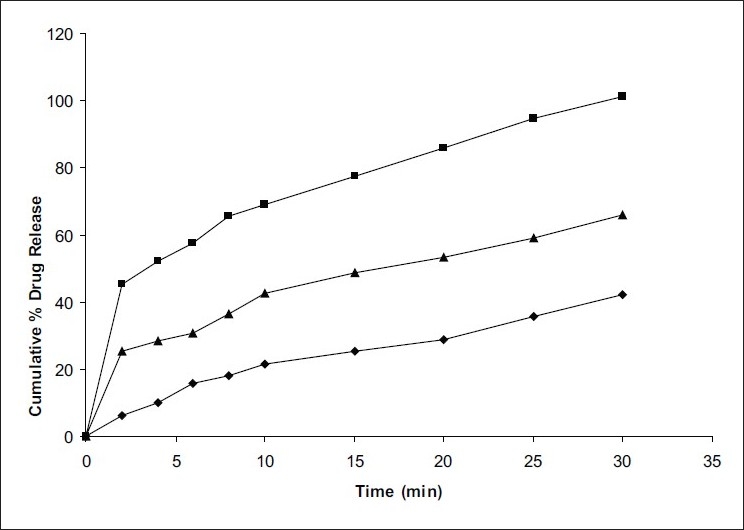
*In vitro* cumulative percent drug release versus time profile of promising clonazepam formulations Plot showing cumulative percent drug release in pH 6.8 phosphate buffer from control formulations (-♦-); promising F3 formulation (-▪-); conventional commercial tablet formulation CF (-▲-).

**TABLE 3:** *IN VITRO* DISSOLUTION PARAMETERS IN PH 6.8 PHOSPHATE BUFFER

Formulation Code	D_5_ (%)	D_10_ (%)	D_15_ (%)	DE_10min_ (%)	t_50%_ (min)	t_70%_ (min)	t_90%_ (min)
F0	12.5	22.0	25.0	22.76	>30	>30	>30
F3	54.0	69.0	77.0	28.91	3.5	10.5	22.5
CF	29.5	42.5	48.5	33.41	16.4	>30	>30

F_0_ is control formulation, F_3_ is promising fast dissolving tablet formulation, CF is conventional commercial tablet formulation, D_5_ is percent drug released in 5 min, D_10_ is percent drug release in 10 min, D_15_ is percent drug release in 15 min, DE_10min_ is dissolution efficiency at 10 min, t_50%_ is time for 50% drug dissolution, t_70%_ is time for 70% drug dissolution, t_90%_ is time for 90% drug dissolution

IR spectroscopic studies indicated that the drug is compatible with all the excipients. The IR spectrum of F_3_ showed all the characteristic peaks of CZ, thus confirming that no interaction of drug occurred with the components of the formulation. Short-term stability studies of the above formulation indicated that there are no significant changes in drug content and *in vitro* dispersion time at the end of 3 mo period (*P*<0.05).

Polynomial equation for 3^2^ full factorial design with two independent variables i.e., proportion of CP (X_1_) and proportion of MCC (X_2_), at three levels is[14], Y=b_0_+b_1_X_1_+b_2_X_2_+b_12_X_1_X_2_+b_11_X_1_^2^+b_22_X_2_^2^, where Y is the dependent variable, b_0_ arithmetic mean response of nine batches, and b_1_ estimated coefficient for factor X_1_. The main effects (X_1_ and X_2_) represent the average results of changing one factor at a time from its low to high value. The interaction term (X_1_X_2_) shows how the response changes when two factors are simultaneously changed. The polynomial terms X_1_^2^ and X_2_^2^ are included to investigate non-linearity.

The equation derived for *in vitro* dispersion time of the factorial formulations is, Y_1_= 20.44−5.42 X_1_−4.92X_2._ The negative sign for coefficients of X_1_ and X_2_ indicate that as the concentration of disintegrants increases, *in vitro* dispersion time decreases. Validity of this Eqn was verified by designing two extra design check point formulations (C_1_ and C_2_) and determining the *in vitro* dispersion time. The *in vitro* dispersion time values predicted from the equation for these formulations are 30.78 and 10.41 s, whereas those observed from experimental results are 31.0 and 9.60 s, respectively. The closeness of the predicted and observed values for C_1_ and C_2_ in the method indicates validity of derived equation for the dependent variable (*in vitro* dispersion time). The computer generated response surface and contour plots for the dependent variable are shown in figs. 2 and 3, respectively.

**Fig. 2:**
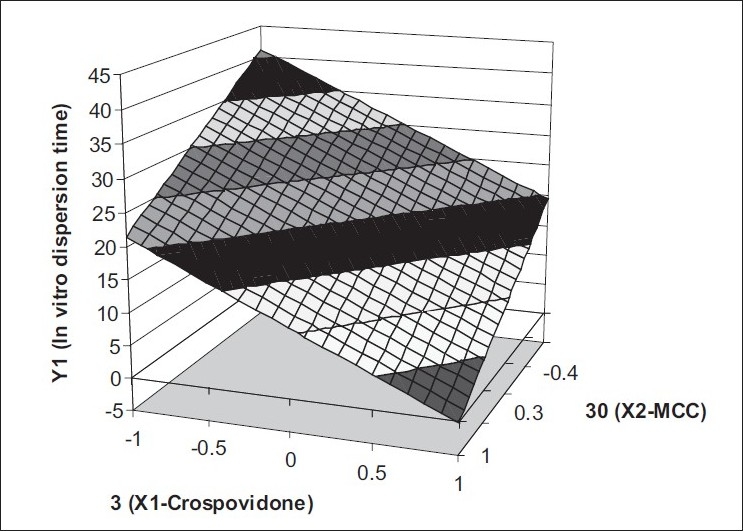
Response surface plot of factorial variables on *in vitro* dispersion time Response surface plot showing effect of factorial variables on *in vitro* dispersion time. The shaded regions indicate the range of response variables, Y1 (*in vitro* dispersion time)

**Fig. 3:**
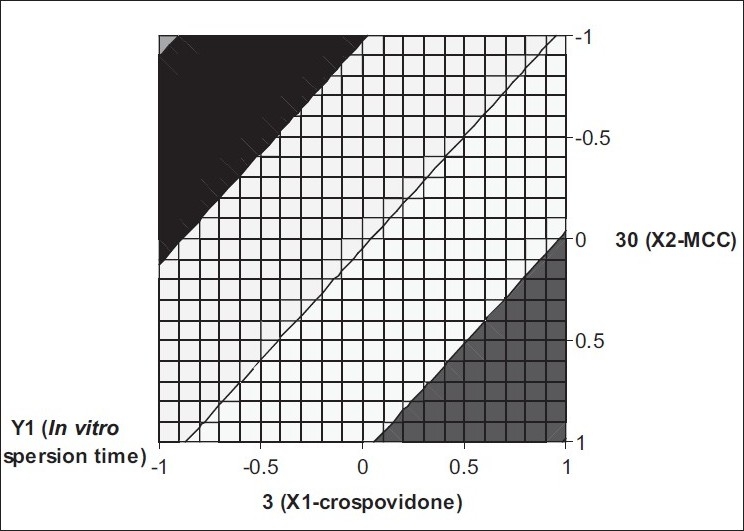
Contour plot of factorial variables on *in vitro* dispersion time The shaded regions indicate the range of response variables, Y1 (in vitro dispersion time)

The results of the 3^2^ full factorial design revealed that the amount of crospovidone and microcrystalline cellulose significantly affect the depended variable, *in vitro* dispersion time. It is thus concluded that by adopting a systematic formulation approach, an optimum point can be reached in the shortest time with minimum efforts.
